# Supplementary material for: The Malaria-Protective Human Glycophorin Structural Variant DUP4 Shows Somatic Mosaicism and Association with Hemoglobin Levels
Source: Am J Hum Genet. 2018 Nov 1;103(5):769–76. doi: 10.1016/j.ajhg.2018.10.008 (PMC6218809; doi:10.1016/j.ajhg.2018.10.008)
Supplement: Document S1. Figures S1–S3 and Tables S1 and S2 [file mmc1.pdf]

The American Journal of Human Genetics, Volume *103*

Supplemental Data

The Malaria-Protective Human Glycophorin Structural Variant DUP4 Shows Somatic Mosaicism and Association with Hemoglobin Levels

Walid Algady, Sandra Louzada, Danielle Carpenter, Paulina Brajer, Anna Färnert, Ingegerd Rooth, Billy Ngasala, Fengtang Yang, Marie-Anne Shaw, and Edward J. Hollox

SUPPLEMENTARY MATERIAL

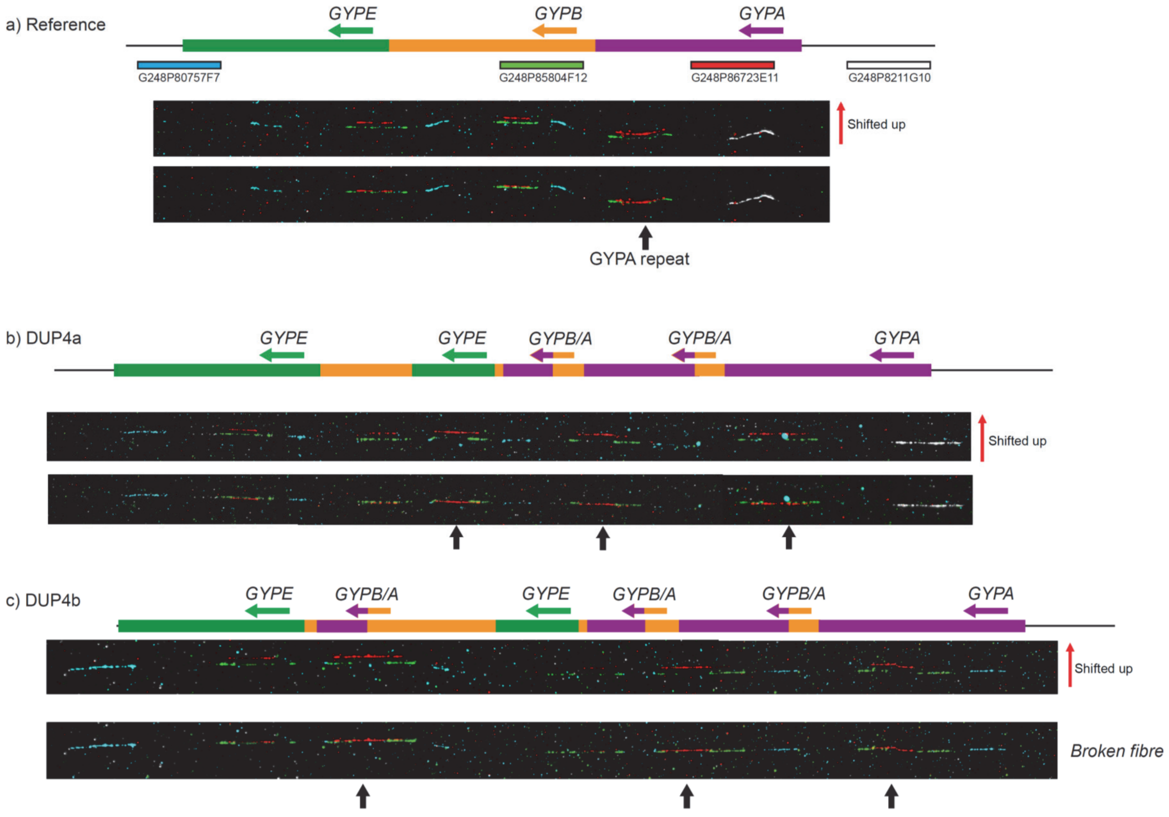

Figure S1 Fiber FISH analysis of the DUP4 heterozygote sample HG02554 using a GYPA-repeat fosmid probe

a) An example DNA fiber from the reference haplotype. The names, relative position and colors of the fosmid probes are indicated above the fiber. The upper image is generated from the lower image, shifting up the red signal to show the gap in the green fosmid signal specific to the GYPA repeat.

b) An example DNA fiber from the DUP4a haplotype. The Leffler model of the DUP4 haplotype is indicated above the fiber, and arrows indicate GYPA repeat units or GYPA partial repeat units. Note that the complete DUP4 pattern consists of two images of the same fiber stitched together.

c) An example DNA fiber from the DUP4b haplotype. Our model of the DUP4b somatic variant haplotype is indicated above the fiber, and arrows indicate GYPA repeat units or GYPA partial repeat units. Note that for this experiment although several images were captured, none were from a full-length fiber with both distal (white) and proximal (blue) fosmid signals. In this fiber, the white and GYPA signals were missing from the distal end of the fiber.

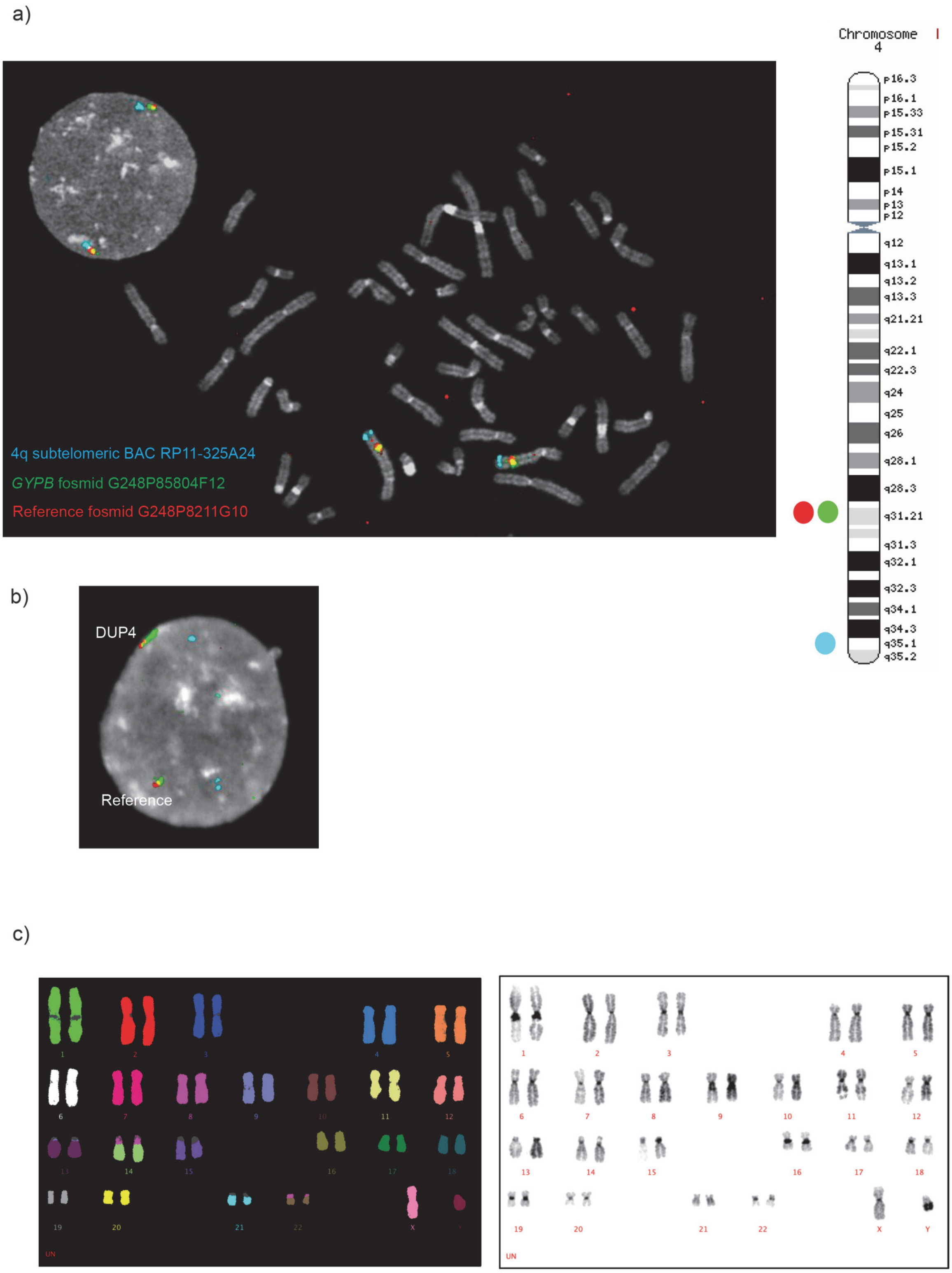

Figure S2 FISH analysis of the DUP4 heterozygote sample HG02554

a) Metaphase-FISH and interphase-FISH analysis of the glycophorin region. Probe locations shown in the ideogram on the right, and shown in more detail in figure 1.

b) An interphase-FISH magnified image, showing that the DUP4 variant can be distinguished by a larger signal from the green probe mapping to the GYPB repeat.

c) Multiplex FISH (left) and DAPI-stained banding (right) of HG02554 cell line, showing no major rearrangements or aneuploidies.

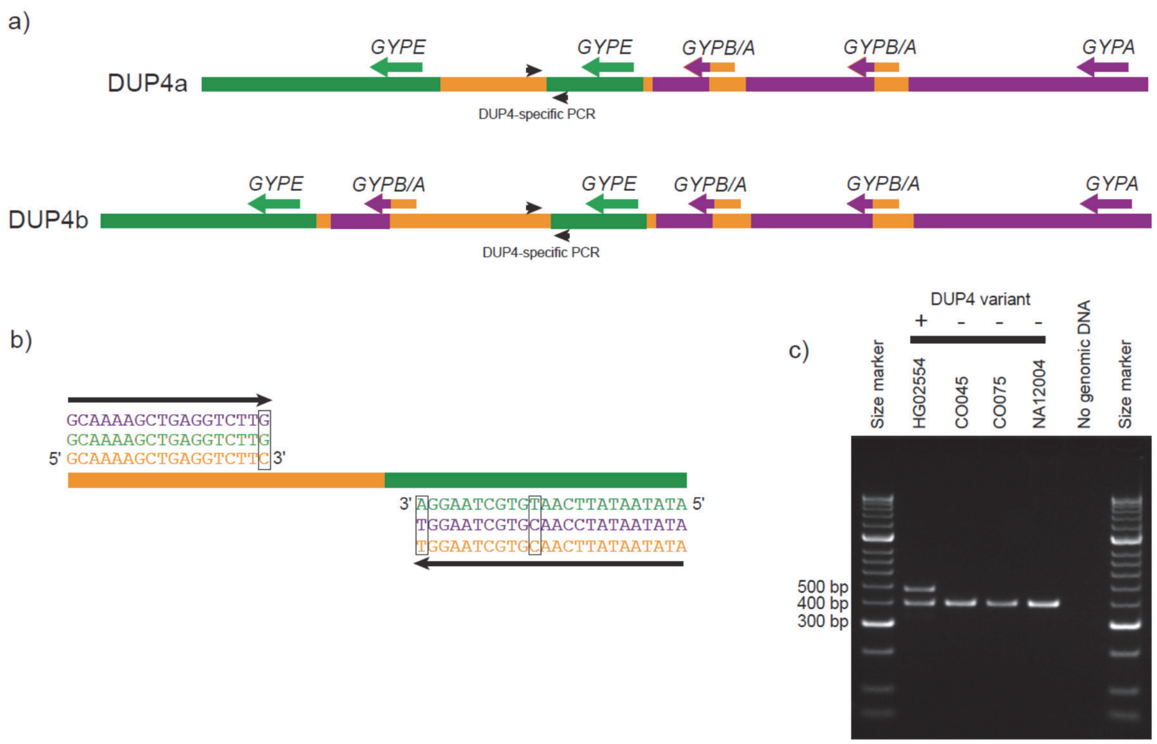

Figure S3 **Genotyping using DUP4 junction fragment PCR**

a) PCR primer design strategy for a DUP4-specific PCR

b) PCR primers incorporating mismatches to ensure paralogue specificity across the B-repeat/E-repeat breakpoint.

c) An image of an ethidium bromide-stained agarose gel showing PCR products generated by the DUP4 junction fragment PCR (~500bp) and a co-amplified control PCR product (~400bp). A known DUP4 heterozygous positive sample (HG02554) and three DUP4 negative samples are shown.

Table S1 1000 Genomes control samples for DUP4 genotyping assay

**Sample**	**Population**	**Known structural variant carried**	**DUP4 assay result**
HG02554	ACB	DUP4	Positive
NA18646	CHB	DUP14	Negative
NA19084	JPT	DUP28	Negative
HG02679	GWD	DUP7	Negative
NA18625	CHB	DUP2	Negative
NA19360	LWK	DUP3	Negative
NA19474	LWK	DUP3	Negative
HG02716	GWD	DEL7	Negative
NA19084	JPT	DUP28	Negative
NA19085	JPT	None	Negative
NA19190	YRI	DEL2	Negative
NA19777	MEX	None	Negative
NA19818	ASW	DEL2	Negative
HG02585	GWD	DUP5	Negative
NA19120	YRI	DEL1	Negative
HG04039	STU	DEL6	Negative
HG03837	STU	DUP24	Negative
NA12004	CEU	None	Negative
NA11839	CEU	None	Negative
NA07034	CEU	None	Negative
NA07055	CEU	None	Negative
NA12814	CEU	None	Negative
NA12005	CEU	None	Negative

Table S2 **Primer sequences used in this study**

**Primer name**	**Primer sequence 5'-3'**
Specific_glycophorinE_F	TGTATTCCAGTTGGTGGATATTTC
Specific_glycophorinE_R	ATGGGCTTTCAAGAACATTTCT
DUP4F2	GCAAAAGCTGAGGTCTT<u>C</u>
DUP4R2	ATATAATATTCAATGTGCTAAGG<u>A</u>
rs186873296F	GAATTCGTTCTTGAAGGCAATT
rs186873296R	GGAGCTTAAGCTTAAATGCCAAGT

Underlined nucleotides indicate that a linked nucleic acid (LNA) nucleotide was used at that position to increase paralog-specificity.
